# Implantable cardiac monitors: artificial intelligence and signal processing reduce remote ECG review workload and preserve arrhythmia detection sensitivity

**DOI:** 10.3389/fcvm.2024.1343424

**Published:** 2024-01-23

**Authors:** Giovanni Bisignani, Jim W. Cheung, Roberto Rordorf, Valentina Kutyifa, Daniel Hofer, Dana Berti, Luigi Di Biase, Eimo Martens, Vincenzo Russo, Paolo Vitillo, Marlies Zoutendijk, Thomas Deneke, Irina Köhler, Jürgen Schrader, Gaurav Upadhyay

**Affiliations:** ^1^ Department of Cardiology, Ospedale Civile Ferrari, Castrovillari, Italy; ^2^ Division of Cardiology, Weill Cornell Medicine, New York, NY, United States; ^3^ Department of Cardiology, IRCCS Policlinico San Matteo, Pavia, Italy; ^4^ Clinical Cardiovascular Research Center, University of Rochester, Rochester, NY, United States; ^5^ Department of Cardiology, University Hospital Zurich, Zurich, Switzerland; ^6^ Department of Cardiology, Jessa Ziekenhuis, Hasselt, Belgium; ^7^ Arrhythmia Services, Albert Einstein College of Medicine at Montefiore Health System, New York, NY, United States; ^8^ Department of Cardiology, Klinikum rechts der Isar, Technical University Munich, Munich, Germany; ^9^ Department of Cardiology, University Vanvitelli, Monaldi Hospital, Napoli, Italy; ^10^ Department of Cardiology, Azienda Ospedaliera di Rilievo Nazionale e di Alta Specialità San Giuseppe Moscati, Avellino, Italy; ^11^ Department of Cardiology, Admiraal de Ruyter Ziekenhuis, Goes, Netherlands; ^12^ Department of Cardiology, Rhön Clinic Campus Bad Neustadt, Bad Neustadt a. d. Saale, Germany; ^13^ Biotronik SE & CO. KG, Berlin, Germany; ^14^ Center for Arrhythmia Care, University of Chicago Medicine, Chicago, IL, United States

**Keywords:** implantable cardiac monitor, remote monitoring, cardiac arrhythmia, employee workload, artificial intelligence

## Abstract

**Introduction:**

Implantable cardiac monitors (ICMs) provide long-term arrhythmia monitoring, but high rates of false detections increase the review burden. The new “SmartECG” algorithm filters false detections. Using large real-world data sets, we aimed to quantify the reduction in workload and any loss in sensitivity from this new algorithm.

**Methods:**

Patients with a BioMonitor IIIm and any device indication were included from three clinical projects. All subcutaneous ECGs (sECGs) transmitted via remote monitoring were classified by the algorithm as “true” or “false.” We quantified the relative reduction in workload assuming “false” sECGs were ignored. The remote monitoring workload from five hospitals with established remote monitoring routines was evaluated. Loss in sensitivity was estimated by testing a sample of 2000 sECGs against a clinical board of three physicians.

**Results:**

Of our population of 368 patients, 42% had an indication for syncope or pre-syncope and 31% for cryptogenic stroke. Within 418.5 patient-years of follow-up, 143,096 remote monitoring transmissions contained 61,517 sECGs. SmartECG filtered 42.8% of all sECGs as “false,” reducing the number per patient-year from 147 to 84. In five hospitals, nine trained reviewers inspected on average 105 sECGs per working hour. This results in an annual working time per patient of 83 min without SmartECG, and 48 min with SmartECG. The loss of sensitivity is estimated as 2.6%. In the majority of cases where true arrhythmias were rejected, SmartECG classified the same type of arrhythmia as “true” before or within 3 days of the falsely rejected sECG.

**Conclusion:**

SmartECG increases efficiency in long-term arrhythmia monitoring using ICMs. The reduction of workload by SmartECG is meaningful and the risk of missing a relevant arrhythmia due to incorrect filtering by the algorithm is limited.

## Introduction

Implantable cardiac monitors (ICMs) are miniaturized devices that are implanted subcutaneously to monitor the heart rhythm over extended periods of time. ICMs record single-channel subcutaneous ECGs (sECG) of detected arrhythmias which can be transmitted via remote monitoring to clinicians. ICMs are used in various challenging diagnostic areas (1) and the ICM follow-up burden is significant (2).

In all diagnostic tools with imperfect accuracy, there is a conflict between sensitivity, for ICMs that is the ability to detect any occurrence of arrhythmia, and specificity, the ability to record only true arrhythmic events. Enhancing one aspect inevitably results in a compromise with the other. ICMs are designed to be highly sensitive. However, the disadvantage of this design choice is that a relatively large number of sECG recordings need review, a significant portion of which are false detections, resulting in additional workload for event classification.

While certain published studies claim small numbers of false detections for some ICMs, these have mostly been derived in a carefully selected clinical setting or a bench test (3). Real-world registries show that up to 80% of all detections are not true arrhythmias (4). Therefore, ICM manufacturers try to reduce false detections by improving detection algorithms (5, 6).

The new BioMonitor IV ICM uses the “SmartECG” algorithm integrated in the remote monitoring platform to recognize incorrectly detected events by advanced signal processing tools and artificial intelligence. The clinician viewing the sECGs can reduce the workload by ignoring sECGs filtered by the SmartECG algorithm as likely false detection.

The present study utilized a large sample of sECGs snapshots from real-world data-sets to assess the workload reduction for reviewing remotely transmitted sECGs after pre-filtering by the SmartECG algorithm and the proportion of true arrhythmias inappropriately filtered by the SmartECG algorithm.

## Materials and methods

### Data sources and patient populations

Data for the present study were pooled from three clinical projects: the CERTITUDE registry, which is a real-world database of U.S. health insurance data and remote monitoring data (7, 8), the BIO|MASTER.BIOMONITOR III study (9, 10) (NCT04025710), and the BIO|STREAM.ICM study (9, 10) (NCT04075084; the latter two with patients from eight European countries and Australia). In all three projects, ethics committee or competent authority approvals were obtained and patients consented to the scientific use of their data.

All patients from these projects were included in the present analysis if they had a BioMonitor IIIm ICM device, except for patients without a reported ICM indication and patients who had contributed sECGs to the training of SmartECG artificial intelligence algorithm for atrial fibrillation (AF) recognition. The observation period included first remote message transmission to the last remote message, the subject's study exit or data collection on 14 October 2022, whichever was earliest.

Indications for ICMs were categorized as syncope/pre-syncope, cryptogenic stroke, atrial fibrillation (AF) monitoring in patients with known AF, palpitations, and others.

### Devices

BioMonitor IV (Biotronik SE & Co. KG, Berlin, Germany) automatically detects arrhythmias labelled as AF, bradycardia (brady), pause, or tachycardia (tachy). It also records sECGs triggered by a sudden rate drop (SRD), manually by the patient (using a small external trigger device), and at a programmed time of the day (“regular sECG”). In the default setting, all detections are enabled except SRD. Using remote monitoring technology (Biotronik Home Monitoring®), the ICM sends a message to the Home Monitoring Service Center (HMSC) every day, containing up to six sECGs.

When this project was initiated, BioMonitor IV was not released to the market and therefore sECGs from BioMonitor IIIm were used, which is equivalent in nearly all aspects concerning this analysis. The only difference relevant to this analysis is that BioMonitor IV transmits a regular sECG every day along with up to five arrhythmia sECGs, while BioMonitor IIIm only transmits a regular sECG at larger pre-programmed intervals, leaving room for six sECGs of arrhythmia on all other days. To obtain an equal data sample which the BioMonitor IV would have provided in the patient cohort, we removed the sixth arrhythmia sECG from each transmission in the BioMonitor IIIm data set.

### Home Monitoring Service Center and workflow

The HMSC supports an event-driven workflow. While the ICM transmits data every day, only transmissions with events are flagged clinical review. Depending on the patient's clinical indication, the clinician can individually tailor and pre-define different findings (events) as a red or yellow alert. When the event has been acknowledged by the user, the alert flag is removed but the event record remains available. Daily review of alerts is recommended (11), whereas patients without alerts can be ignored.

User-selected alert settings in the HMSC were not available in our data-set; therefore, all detected arrhythmias were assumed to be yellow alerts, as this is the default setting in the HMSC.

### SmartECG algorithm

SmartECG is a software package implemented in the HMSC for the BioMonitor IV ICM. The software evaluates sECGs and labels them as “SmartECG true” (presumed real arrhythmia) or “SmartECG false” (presumed incorrect detection). In rare cases, the algorithm may also fail to reach a conclusion (“no evaluation possible”). SmartECG uses advanced signal processing tools to identify QRS complexes. For the analysis of AF events only, it further uses artificial intelligence, which can be set to different levels of strictness, from very sensitive to very specific. Our analysis uses the nominal “balanced” setting. SmartECG does not analyze SRD, patient triggered, and regular sECGs.

### Sensitivity of SmartECG

To estimate the reduction in sensitivity of BioMonitor IV with the SmartECG algorithm compared to BioMonitor IIIm, we selected a sample of 2000 ICM-detected episodes, comprising of 500 each from AF, brady, pause, and tachy sECGs. This was achieved step-by-step: the first sECGs from all patients with at least one sECG of the desired type were first selected, then the second sECGs, third, and so on, until 500 sECGs of each type were available. Only one sECG per day and patient was used.

A clinical board of physicians adjudicated the appropriateness of ICM detections. If the first two board members agreed, their adjudication was valid; if not, a third vote was required and the majority vote was accepted. The sensitivity of SmartECG was defined as the percentage of sECGs with true arrhythmia according to the clinical board that are not filtered by SmartECG.

As a measurement of the uncertainty of the board's decision, we indicate the proportion of sECGs that were not equally classified by the first two adjudicators. Furthermore, to assess the clinical relevance of sECGs that were inappropriately filtered by SmartECG, we checked whether the patients with such sECGs had additional sECGs of the same type that were not filtered by SmartECG.

### Workload estimation

To quantify a generalizable review burden, we measured the time spent on daily HMSC checks for BioMonitor patients in clinical practice from five hospitals. All sites had established remote monitoring routines and had between 350 and 1,600 cardiac implantable electronic devices under remote monitoring. Because the HMSC workflow is similar for all Biotronik ICM devices, this analysis also includes older BioMonitor generations. During clinical routine, trained reviewers recorded the duration of their remote monitoring sessions dedicated to BioMonitor devices and the amount of sECGs screened in this time. Since arrhythmias are the only alerts that ICMs transmit, we divided the total working time by the total number of inspected sECGs. Based on this result and the observed number of sECGs, we calculated the follow-up workload in hours per patient-year of follow-up and estimated the reduction in workload with SmartECG. For the latter case, we assumed that the reviewer ignored all sECGs filtered by the algorithm. In addition to the four types of arrhythmia checked by SmartECG, this estimation also included SRD, patient triggered and regular sECGs, if they were inspected. The recorded time does not include time to contact patients, if clinically relevant results were found.

### Statistics

Continuous data are presented as mean value ± standard deviation and as absolute and relative frequencies. Nominal data were compared with the exact Fisher test. A *P*-value <0.05 was considered statistically significant. The analyses were performed using the R (R Development Core Team, https://www.R-project.org/) statistical software.

## Results

### Patients

Of 725 CERTITUDE registry patients with a BioMonitor IIIm device at the time of data collection, 358 had no ICM indication reported and 198 had contributed to the training of the artificial intelligence algorithm. After excluding these patients, the present analysis included 169 CERTITUDE registry patients, 184 BIO|STREAM.ICM study patients, and 15 BIO|MASTER.BIOMONITOR III study patients, for a total of 368 patients.

Mean age of the patients was 66.1 ± 15.1 years and 38.0% were women. Most patients had an indication for syncope or pre-syncope (41.6%) or for cryptogenic stroke (31.0%) (Table 1). Patients were followed for a total of 418.5 patient-years.

**Table 1:** Number of sECGs per day/year according to ICM indication without/with SmartECG.

ICM indication	Patients *N* (%)	Patient-years of follow-up	No SmartECG		With SmartECG	
			sECG N/day	sECG N/year	sECG N/day	sECG N/year
Syncope or pre-syncope	153 (41.6%)	151.6	0.58	212	0.32	117
Cryptogenic stroke	114 (31.0%)	135.2	0.22	82	0.11	40
AF management	60 (16.3%)	87.7	0.44	159	0.31	114
Palpitations	24 (6.5%)	28.4	0.24	87	0.15	57
Other	17 (4.6%)	15.6	0.33	119	0.09	34
Total	368	418.5	0.40	147	0.23	84

AF, atrial fibrillation; ICM, implantable cardiac monitor.

### Transmitted sECGs

A total of 143,096 daily remote monitoring transmissions were received during 418.5 patient-years of follow-up, or 341.9 per patient-year (ppy). These transmissions contained 61,517 sECGs, corresponding to 147 sECGs ppy or 0.40 sECGs per patient-day. The largest number of sECGs were obtained in patients with syncope (212 ppy) and patients with AF management (159 ppy) (Table 1).

### Detected arrhythmias and filtering by SmartECG

**Figure 1:**
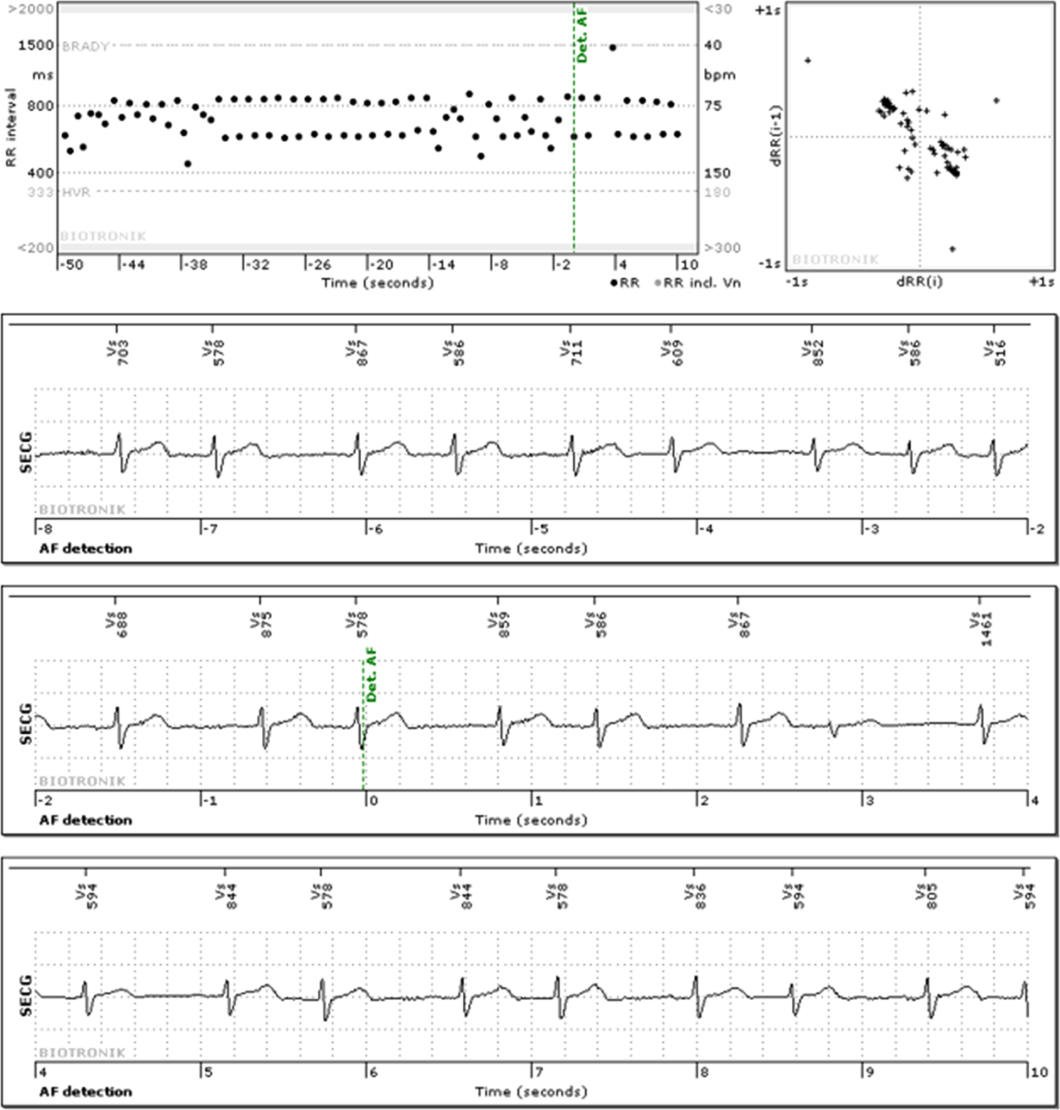
Example of an sECG with an “AF” detection, classified as false detection both by the clinical board and SmartECG.

The most common events detected by the ICM were AF (36.1%) and bradycardia (34.9%) (Table 2). Sudden rate drop was observed least often (1.4%). SmartECG filtered 42.8% of all sECGs (Table 2). An example of an incorrect “AF” detection filtered by SmartECG is shown in Figure 1. The type of arrhythmia recognized by SmartECG as incorrect was most commonly pause (85.6% of events were filtered) and least frequently was bradycardia (28.6% filtered). By using the SmartECG algorithm, the number of sECGs per patient-year was reduced from 147 to 84 (Table 1).

**Table 2:** Proportion of sECGs filtered by SmartECG.

Patients	All sECGs *N* (%)	Filtered sECGs by SmartECG *N* (%)
Total	61,517	26,324 (42.8%)
ICM-detected arrhythmia		
Atrial fibrillation	22,235 (36.1%)	10,268 (46.2%)
Pause	7,507 (12.2%)	6,426 (85.6%)
Bradycardia	21,489 (34.9%)	6,151 (28.6%)
Tachycardia	8,050 (13.1%)	3,479 (43.2%)
Sudden rate drop	867 (1.4%)	0 (0.0%)
Patient triggered sECG	1,371 (2.2%)	0 (0.0%)

ICM, implantable cardiac monitor.

### Estimate of the workload

Nine trained reviewers (2 physicians and 7 nurses or technicians) in five sites inspected an average of 105 sECGs per working hour, equivalent to 34 s for one sECG (Table 3). The number of sECGs per hour of working time varied widely, between 27 and 150 sECGs per hour between hospitals. The two physicians among the 9 operators took more time to inspect the sECGs but the data are not sufficient for a generalizable result. For 100 patients with ICMs, the daily working time to manage the remote monitoring transmissions is calculated as 0.38 h or 23 min: 100 patients multiplied by 0.40 sECGs per patient-day divided by 105 sECGs/hour. This is equivalent to 83 min per patient-year. By using SmartECG, the sECG review workload is reduced to 13.1 min for 100 patients per day or 47.7 min per patient and year.

**Table 3:** Workload per sECG adjudication.

Hospital/Operators	Sessions	Duration per session	Total duration	ECGs	ECG per hour
(1) 1 nurse/technician	5	1:46	8:50	914	103.5
(2) 1 physician	3	0:30	1:30	55	36.7
(3) 1 physician	3	0:30	1:30	40	26.7
(4) 4 nurse/technician	10	0:33	5:39	846	149.7
(5) 2 nurse/technician	8	0:09	1:15	117	93.6
Total 9	29	0:39	18:44	1,972	105.3

### Sensitivity

A total of 708 events were adjudicated by the clinical board as correctly detected. The SmartECG algorithm filtered 2.1% (15/708) of true arrhythmia events, with the rejection rate ranging from 0.0% for bradycardia to 4.4% for AF (Table 4). Accounting for the distribution of arrhythmia types from the complete data set, 2.56% of all true arrhythmias would be rejected by SmartECG (Table 4).

**Table 4:** Sensitivity of SmartECG, the proportion of true arrhythmia filtered by SmartECG.

Arrhythmia type	True arrh.^a^ *N* =	Rejected by SmartECG *N* (%)	Sensitivity of SmartECG	% of sECGs in the total sample^b^	% of true arrh. filtered in the total sample
ICM-detected					
Atrial fibrillation	114	5 (4.4%)	95.6%	36.1%	1.58%
Pause	85	0 (0.0%)	100%	12.2%	0.0%
Bradycardia	279	6 (2.2%)	97.8%	34.9%	0.75%
Tachycardia	230	4 (1.7%)	98.3%	13.1%	0.23%
Sudden rate drop	n.a.	n.a.	100%	1.4%	0.0%
Patient triggered sECG	n.a.	n.a.	100%	2.2%	0.0%
Total	708	15 (2.1%)	97.9%	100%	2.56%

Arrh., arrhythmia; ICM, implantable cardiac monitor; n.a., not applicable.

^a^ Adjudicated by three-member clinical board.

^b^ Data taken from Table 2.

Of the 15 patients who had the 15 episodes of true arrhythmia rejected by SmartECG, 12 patients had episodes of the same type of arrhythmia recognized as correct by SmartECG either before or within 3 days after the “false-negative” cases, one patient had a pause that was not rejected by SmartECG on the same day on which the bradycardia sECG was incorrectly rejected, and the remaining two patients had no further (true positive of false negative) SmartECG evaluations of the same type of arrhythmia until the end of the observation period.

Disagreement between the first two adjudicators was more frequent for AF episodes (8.6%, *n *=* *43) than for other arrhythmia types (0.4% brady [*n *=* *2], 1.8% tachy [*n *=* *9], 2.0% pause [*n *=* *10]) (*P* < 0.001).

## Discussion

The results of this analysis demonstrate SmartECG's ability to reduce the clinical workload of sECG evaluation by approximately 40%, at the cost of filtering of 2.6% of true arrhythmias.

We analyzed the complete set of all sECGs transmitted by remote monitoring from a population of unselected ICM patients within nearly 420 patient-years of follow-up, from a wide range of countries, without selection by device indication. In the sample of more than 64,000 sECGs snapshots, SmartECG classified 42.8% as incorrectly detected. This rejection (clinical workload reduction) was largest for pause (85%) and lowest for bradycardia (29%).

### Safety: sensitivity

A sample of sECGs adjudicated by a board of three physicians allowed us to estimate the reduction of sensitivity caused by the use of SmartECG algorithm. Of all true arrhythmias, 2.6% were rejected by SmartECG. This evaluation depended on the type of arrhythmia, with the highest rejection rate observed for AF episodes (4.4%).

Any algorithm can only be as good as the gold standard against which it is tested. It should be acknowledged that it is occasionally difficult to differentiate AF from a rhythm with frequent atrial ectopies in a single-lead ECG. This applies to the clinical reviewer, the SmartECG algorithm, and the expert board alike. In 8.6% of all AF-related sECGs adjudicated by the board, the first two adjudicators disagreed, but only in 1.4% for pause, brady, and tachy sECGs pooled (*P* < 0.001). The board's error rate, which is of course not quantifiable, places a theoretical upper limit on our assessment of the algorithm's performance. Therefore, the rejection of true AF episodes by SmartECG may actually be lower than the 4.4% identified in this analysis.

To assess the potential clinical impact of erroneously filtered sECGs, we examined patients with such episodes individually. Twelve of 15 patients with a falsely filtered sECG had the same arrhythmia type recognized correct by SmartECG before or immediately after sECG rejection (within 3 days), and one patient had a pause event (classified as correct by SmartECG) on the day of inappropriately rejected bradycardia sECG. This suggests that a relevant share of patients with falsely filtered sECG may receive their diagnoses despite some algorithm errors.

Although the reduction of sensitivity it thus limited and may be considered acceptable, further improvements of specificity and sensitivity are required.

### Efficacy: review time

As arrhythmia sECGs are the only alerts that ICMs generate, and patients without alerts are ignored during daily remote monitoring checks, we estimate that the *relative* reduction of workload by SmartECG is 42.8%, equal to the percentage of filtered sECGs. In order to estimate the *absolute* workload reduction through SmartECG, we determined the number of sECGs that a trained reviewer can review per hour in clinical practice in different clinics with an established remote monitoring workflow, in the remote monitoring platform of the manufacturer of the devices we studied. We found a mean workload for a single patient per year of approximately one and a half hours without and 50 min with SmartECG.

Seiler et al. (2) have published a comprehensive analysis of the remote monitoring workload for CIEDs, including ICMs. They reported ≈37 transmissions for ICMs per patient-year, with each transmission requiring ≈12 min of working time. In our sample of Biotronik ICM devices, 342 transmissions were received per patient-year, but many of them did not contain an sECG and therefore did not require attention. In the workflow that the Biotronik Home Monitoring platform supports, switching between patients, transmissions, and alerts is fluent and measuring the “working time per transmission” does not make sense. We found that it takes slightly more than half a minute to assess an sECG (including the time to switch between patients with events), translating into an annual working time of 50 min. In their article, Seiler et al. (2) also reported time for administration, contacting patients, and in-house visits; however, for pure “remote device transmission review”, they estimated a total of 6.7 (Europe) and 8.4 (USA) hours per patient-year, which is an order of magnitude more than our estimate. Due to the complex methodology in their study, we do not claim that our workload estimate can be directly compared to theirs. However, we believe that our results clearly show that they overestimate the total workload for the system we studied.

It is important to remember that the SmartECG algorithm does not analyze ECGs which were manually triggered by the patient. These events can be meaningful even if the ECG is perfectly normal and they must be evaluated by the physician, but they are excluded from our workload estimation.

### Remote vs. in-office follow-up

The SmartECG algorithm is implemented in the remote monitoring platform because it cannot be implemented in the ICM or in the programming device used to interrogate the ICM during a follow-up visit. If patients are traditionally followed by regular in-person visits, the improvement in detection that we describe does not apply. In addition to the shorter time to intervention after asymptomatic arrhythmias (12), these results add to the advantages of utilizing remote monitoring.

### Limitations

Limitations to this analysis include the inability to analyze the user-selected alert settings in the HMSC within our data set and thus the assumption that all arrhythmias are alerts and may have caused an overestimation of the workload of remote monitoring, both with and without SmartECG. It is unclear how the consideration of the selected settings would influence the amplitude of the reduction of the workload by SmartECG. We measured the working time per sECG with the mixture of correct and false detections of the device without SmartECG, and it may be different if the proportion of correct detections increases when SmartECG is applied.

## Conclusion

Biotronik's SmartECG algorithm proves to be a valuable tool that can be seamlessly integrated into the routine monitoring of patients with a BioMonitor IV ICM through remote monitoring. The substantial reduction in workload it offers is meaningful, and the algorithm's risk of erroneously rejecting a relevant arrhythmia is minimal.

## Data Availability

The datasets presented in this article are not readily available because the data may contain information that would betray critical intellectual property of the sponsor. Requests to access the datasets should be directed to Jürgen Schrader, juergen.schrader@biotronik.com.
